# Heterogeneous irradiated-pristine polyethylene nanofiber junction as a high-performance solid-state thermal diode

**DOI:** 10.1038/s41598-021-85140-6

**Published:** 2021-03-11

**Authors:** Xiao Luo, Yuxuan Luan, Yutian Cai, Sheng Shen

**Affiliations:** Department of Mechanical Engineering, Carnegie Mellon University, Pittsburgh, PA 15213 USA

**Keywords:** Mechanical engineering, Nanowires, Applied physics

## Abstract

In this work, we demonstrate two types of heterogeneous irradiated-pristine polyethylene nanofiber junctions, ‘heavily-irradiated-pristine’ (HI-P) and ‘lightly-irradiated-pristine’ (LI-P) junctions, as high-performance solid-state thermal diodes. The HI-P junction rectifies heat flux in a single direction, while the LI-P junction shows dual-directional rectification under different working temperatures. We accurately model the phase transition of polyethylene nanofibers with a finite temperature range rather than a step function. The finite-temperature-range model suggests that the rectification factor increases with temperature bias and there is a minimum threshold of temperature bias for notable rectification. Besides, the finite-temperature-range model shows better prediction for the heat flow data from experiments, while the step function model tends to overestimate the rectification performance around the optimal length fraction of irradiation. Although both the models show that an optimal rectification occurs when the interface temperatures in the forward and the reverse biases are equal, the optimized rectification factor is determined by the temperature bias and the temperature range of phase transition. This work elucidates the influence of both the temperature bias and the temperature range of phase transition on thermal rectification performance, which could incredibly benefit the evaluation and design of thermal diodes.

## Introduction

Nonlinear heat transfer has attracted significant interest from heat transfer, thermal physics, and energy communities for new thermal transport phenomena beyond those achieved by thermal resistors and capacitors^1^. Nonlinear thermal components, such as thermal regulators, switches and diodes, are favored by heat transfer engineers for active thermal control^1^. As the counterpart of a diode in electronics, a thermal diode is a two-terminal device that shows asymmetric thermal conductance, transporting heat like a conductor if forward-biased but blocking heat flow like an insulator if reverse-biased, i.e., rectifying heat flow^1^. Theoretical study of heat conduction in 1D nonlinear chains reveals that transport property of the chain is correlated with external potential function and can be altered by changing the temperature of the heat baths^2^. A solid-state thermal rectifier is designed by matching phonon bands if forward-biased but mismatching phonon bands if reverse-biased^2^. Later, the match/mismatch mechanism is found to be effective to realize thermal rectification in anharmonic lattices with a mass gradient in the Fermi-Pasta-Ulam model^3^ and a junction of two nonlinear lattices in the Frenkel-Kontorova model^4,5^. The Thermal rectification has been realized mainly based on heat conduction^1,6–22^, besides phase-change material^23^, natural convection^1^, radiation^24^, evaporation and condensation^25^. Among these mechanisms, conduction-based thermal rectification shows great promise for phononics^26^ and have been widely studied with nanoscale materials^6,11,15,18–22^ as well as bulk materials^8–10,12,16,17^ via temperature-controlled locomotion^10^, mass loading^11^, heterogeneous junctions^6,8,9,12,14–18,23^ and asymmetric geometry^7,13,16,18–22^. Especially, thermal diodes using heterogenous junctions is more advantageous than others because they can achieve a relatively high rectification within a narrow temperature range when using phase transition materials. Theoretically, Go and Sen^27^ proved that the heterogeneous junction of materials with different temperature-dependent thermal conductivities inherently renders thermal rectification. Dames^28^ analyzed the thermal rectification of bulk material junctions and studied the influence of thermal resistance ratio of the two segments on the rectification performance. Yang et al.^29^ studied the influence of the vertex angle and sample length of graphene on the thermal rectification performance in a triangular asymmetric geometry. Sawaki et al.^16^ numerically analyzed the influence of the length ratio and cross-sectional area on the rectification performance. Hu et al.^14^ found that the thermal rectification performance of a heterogeneous junction can be enhanced by a series circuit of single thermal rectifiers. Zhang et al.^15^ and Cottrill et al.^30^ studied the junctions based on the materials with and without phase transition and the optimization condition among thermal switching ratio of the phase transition material, thermal conductivity of the non-phase-transition material and junction geometry. Nonetheless, phase transition was simply approximated as a step function of temperatures in these studies. In reality, phase transition of materials could be more complex, for example, occurring within a finite temperature range, in which the step-function model cannot accurately predict the rectification behavior. In this work, we study the rectification effects of the nanoscale thermal diodes based on high-crystallinity polyethylene (PE) nanofibers, which have solid–solid phase transition within 5–10 K^6,31^. We develop a piecewise power function to mathematically model the phase transition and employ the bisection method to determine heat flow. In contrast with the step-function model, the finite temperature range of phase transition shows non-trivial prediction of thermal rectification. We also validate the optimization condition from literature^15,30^ under a finite temperature range of phase transition and discuss the influence of the temperature range on rectification performance.

## Design and calculation

**Figure 1:**
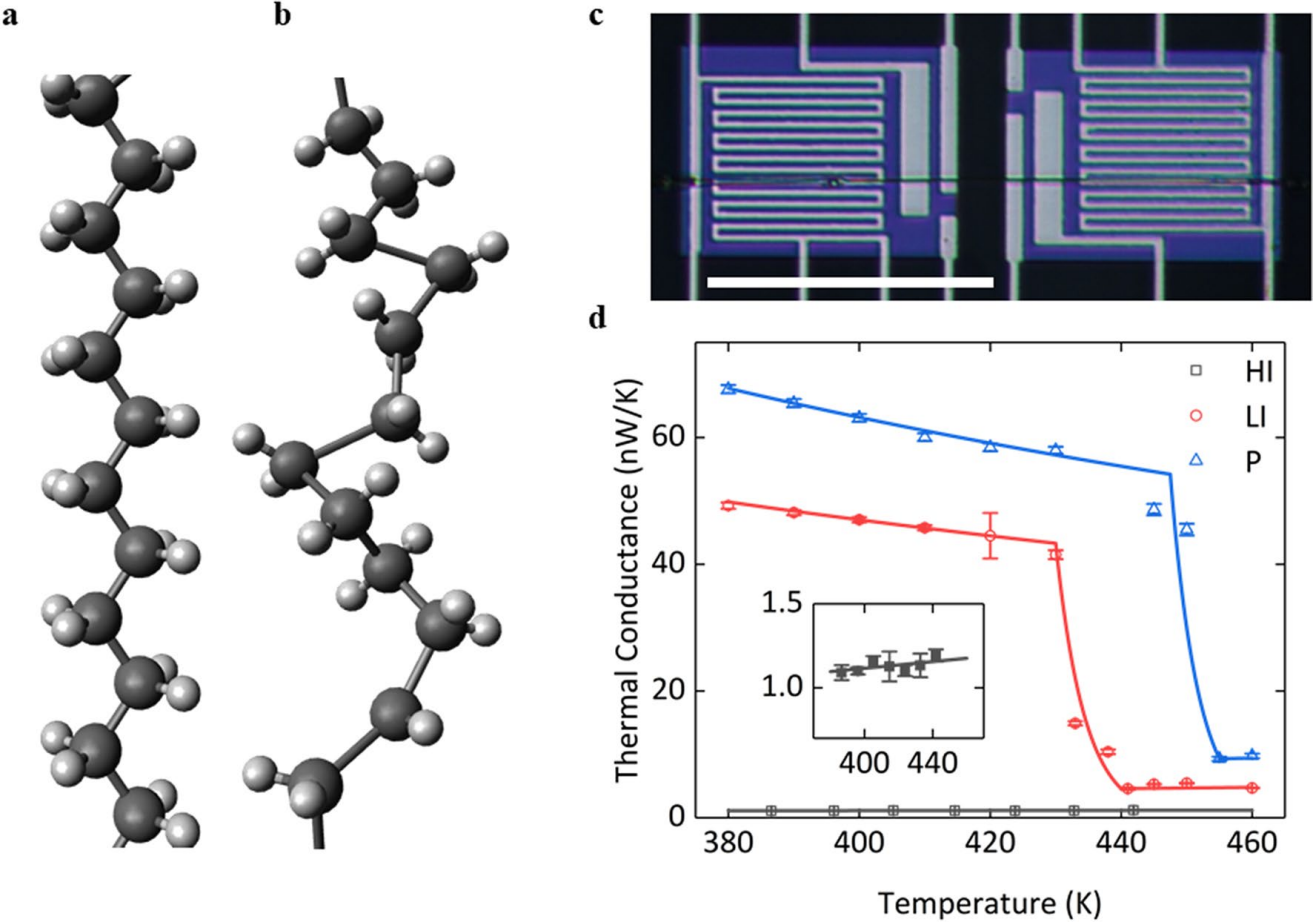
Schematics of a PE molecular chain (**a**) before and (**b**) after phase transition. The molecular chain shows the rotational disorder after phase transition. (**a**) and (**b**) created by Avogadro, version 1.2.0n, https://avogadro.cc/. (**c**) Optical image of a PE nanofiber on a suspended platinum resistance thermometer microdevice. Scale bar, 50 μm. (**d**) Our previous experimental data and theoretical fitting curves of the temperature-dependent thermal conductance of pristine (P), lightly-irradiated (LI) and heavily-irradiated (HI) PE nanofibers. The inset figure shows the linear fitting of experimental data for the HI PE nanofiber.

At room temperature a crystalline PE nanofiber (~ 100 nm in diameter) shows an orthorhombic crystalline structure with extended molecular chains, as shown in Fig. 1a, which leads to an intrinsic high thermal conductivity along the chain direction due to the chain alignment^32^. However, a structural phase transition in the PE nanofiber occurs at temperature above 400 K within a 5–10 K temperature range, in which the highly ordered crystalline structure is switched to a rotationally disordered one, as shown in Fig. 1b. Such a dramatic structural change also corresponds to a sharp decrease of thermal conductivity^31,33,34^. We use electron beam (e-beam) illumination to tune the phase transition behavior of the PE nanofiber, since e-beam irradiation can reduce the molecular orientation and crystallinity of the PE nanofiber^6^. In Fig. 1c, we use a suspended platinum resistance thermometer microdevice to characterize the temperature dependent thermal conductivity of pristine and irradiated PE nanofibers. As shown in Fig. 1d from our previous experimental data^6^, the phase transition of a typical pristine PE nanofiber occurs at around 447.5 K, and thermal conductivity drastically decreases to about one fifth of the original value within a temperature range of 5–10 K. In case of the lightly irradiated (LI) PE nanofiber under ~ 2 s e-beam irradiation with an accelerating voltage of 5 kV, we can still observe the phase transition, but it shifts to a relatively lower temperature of ~ 430 K. However, phase transition no longer exists for the heavily irradiated (HI) sample under a longer e-beam exposure time (> 6 s)^6^.

**Figure 2:**
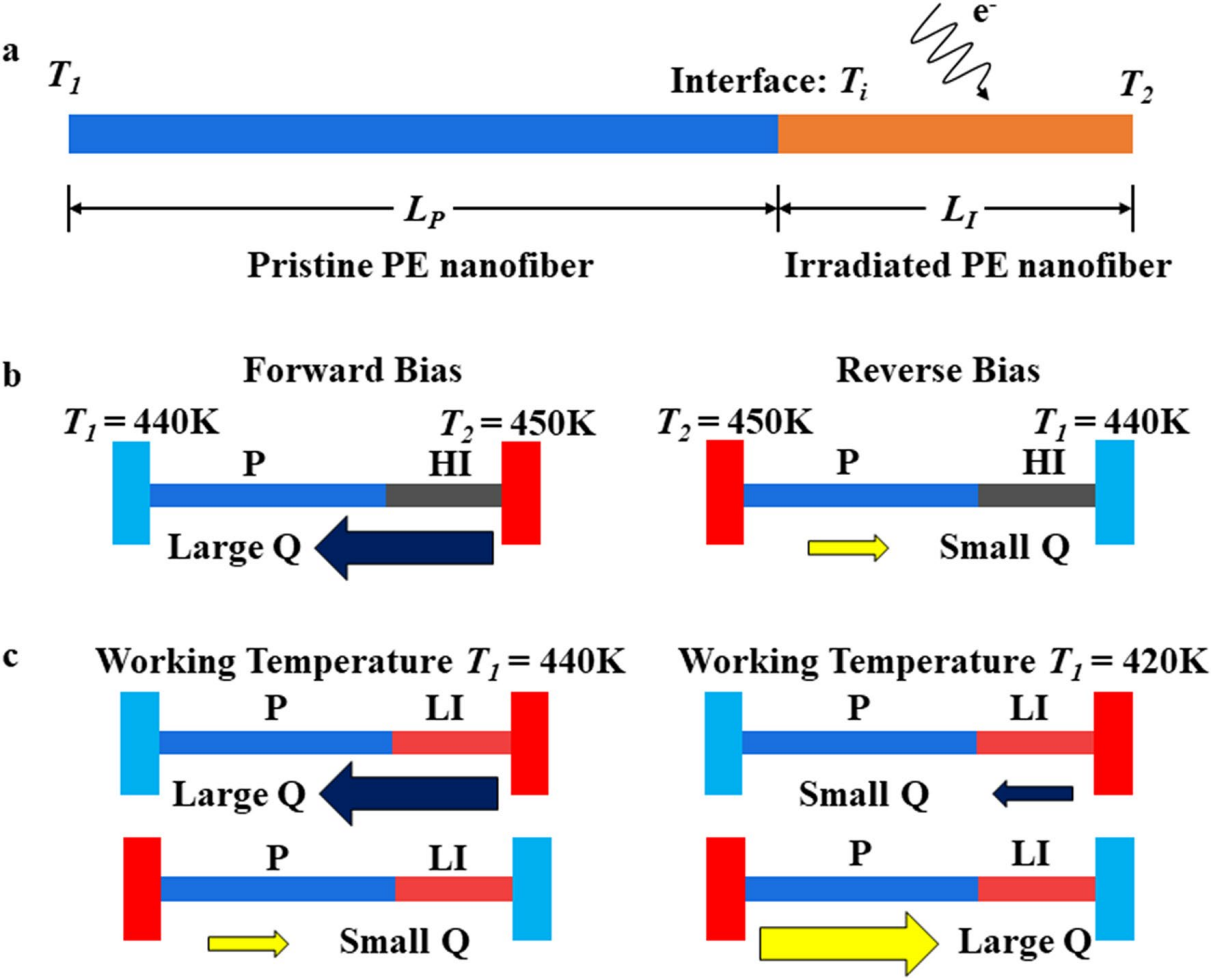
(**a**) Physical model of the ‘irradiate-pristine’ PE nanofiber junction in the forward bias. (**b**) Schematics of the rectification behaviors of the HI-P junction. (**c**) Schematics of the dual-directional rectification of the LI-P junction.

With the distinct thermal transport behaviors between the pristine and the irradiated nanofiber samples, we design heterogeneous “irradiated-pristine” PE nanofiber junctions as nanoscale thermal diodes, as shown in Fig. 2a. Two types of PE nanofiber junctions, heavily irradiated-pristine (HI-P) and lightly irradiated-pristine (LI-P) junctions, can be fabricated by controlling the e-beam exposure time^6^. As shown in Fig. 2b, the pristine part is in the orthorhombic phase with high thermal conductivity if the pristine part is on the cold side, defined as the forward-bias case, and thus heat flow is large. However, phase transition occurs if the pristine part is on the hot side, defined as the reverse-bias case, and the heat flow becomes much less. Thus, the HI-P junction can rectify heat flow in a single direction by suppressing the heat flow in the reverse bias. In contrast, the LI PE nanofiber still retains the structural phase transition at a lower temperature, which enables the dual-directional thermal rectification in the LI-P junction at different working temperatures. As shown in Fig. 2c, phase transition occurs in the pristine part in the reverse bias at a high working temperature (e.g., 440 K), similar with the HI-P junction. However, phase transition also occurs in the LI part in the forward bias at a low working temperature like 420 K, where the reverse heat flow surpasses the forward heat flow and thermal rectification is realized in the opposite direction, leading to “negative rectification”.

Our theoretical analysis is based on the physical model in Fig. 2a, where *T*_1_ is the temperature of the cold end, and *T*_2_ is the temperature of the hot end such that the temperature bias Δ*T* = *T*_2_ − *T*_1_. The interface temperature *T*_*i*_ is then calculated to find out the heat flow through the junction. For a single 1D junction, the heat flow within the pristine part should be equal to the heat flow within the irradiated part,

1 $$Q_{P} = Q_{I} .$$

If we neglect the thermal resistance at the interface, the expression of the heat flow can be written as^28^

2 $$Q_{P} = \int\limits_{{T_{1} }}^{{T_{i} }} {\frac{{\left. {G_{P} (T} \right)}}{{L_{P} }}dT}$$

3 $$Q_{I} = \int\limits_{{T_{i} }}^{{T_{2} }} {\frac{{\left. {G_{I} (T} \right)}}{{L_{I} }}dT}$$

where *G*(*T*) is the thermal conductance for a unit length of a PE fiber. The method of bisection is used to solve *T*_*i*_ that is initially set as the average value of *T*_1_ and *T*_2_. Then, *Q*_*P*_ and *Q*_*I*_ are calculated based on Eqs. (2) and (3), respectively. To fulfill the requirement that the heat flows through the two segments are identical, the iteration method is adopted such that the temperature difference between *T*_1_ and *T*_*i*_ is narrowed down after each iteration by setting the new *T*_*i*_ equal to (*T*_1_ + *T*_*i*_)/2 if *Q*_*P*_ is greater than *Q*_*I*_, or (*T*_*i*_ + *T*_2_)/2 in the other case of *Q*_*P*_ < *Q*_*I*_. In our calculation, we solve the integrals in Eqs. (2) and (3) using Tanh–Sinh quadrature^35^, which is capable of providing accurate results regardless of the smoothness of a function. Similarly, the heat flow in the reverse bias is obtained by swapping the cold end *T*_1_ and the hot end *T*_2_. The rectification factor is defined as

4 $$\varepsilon = \frac{{Q_{fwd} - Q_{rev} }}{{\min \left( {Q_{fwd} ,Q_{rev} } \right)}}.$$

In this study, we consider both the global maximum rectification *ε*_*max*_ and the local maximum rectification at a certain temperature bias *ε*_*max_local*_.

In our model, we linearly fit the temperature dependent thermal conductance of the HI PE nanofiber, while the piecewise function is used to fit the thermal conductances of the pristine and the LI PE nanofibers. The piecewise function consists of three power functions with different powers. Phase transition is assumed to begin at *T*_1_* and end at *T*_2_*, with *G*_*on*_ and *G*_*off*_ being the thermal conductances right before and after phase transition, respectively. The ratio of *G*_*on*_ to *G*_*off*_ is defined as thermal switching ratio. As shown in Fig. 1d, phase transition starts at 447.5 K and 430 K, and ends at 455 K and 440 K for the pristine and the LI nanofibers, respectively.

## Results

**Figure 3:**
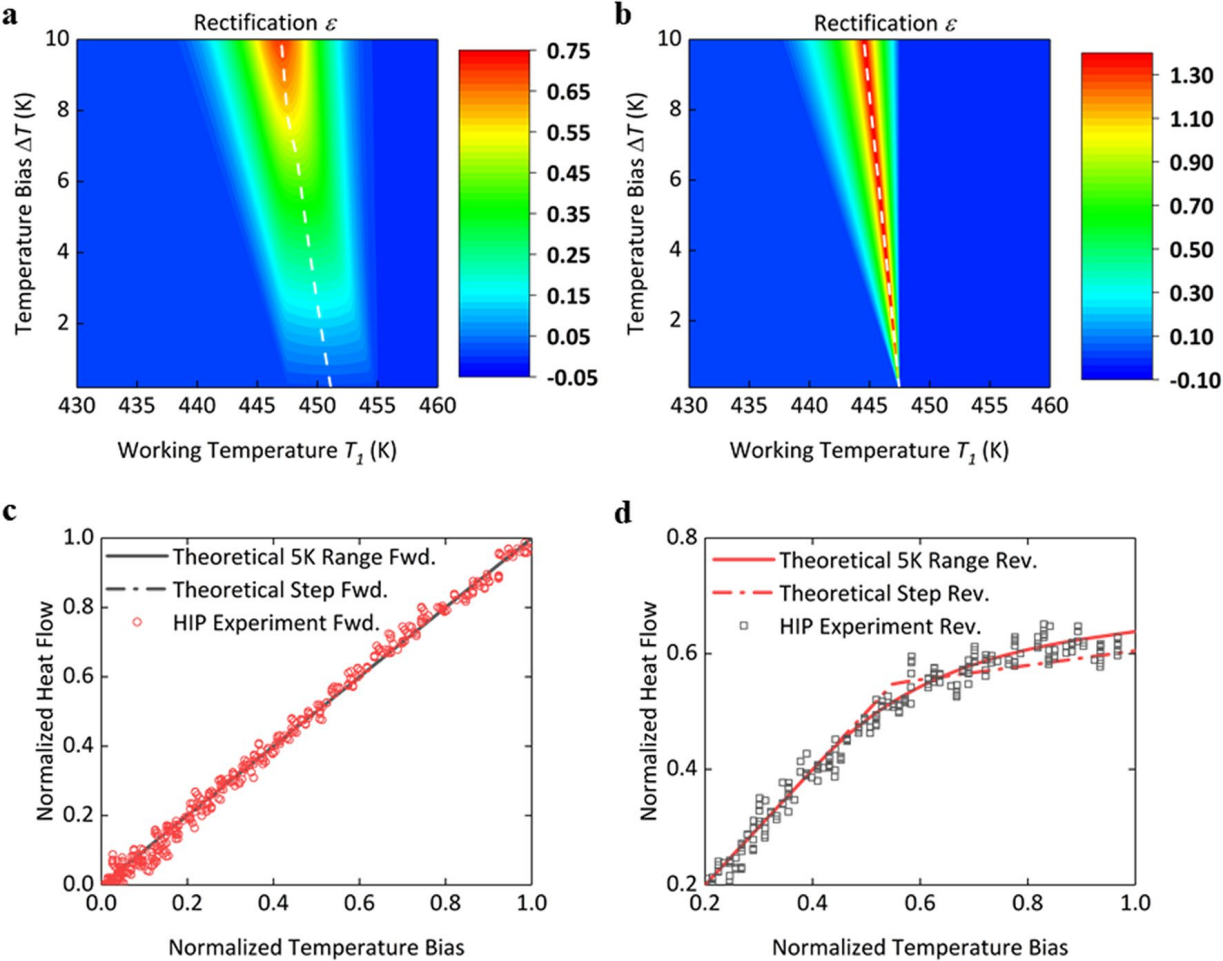
Rectification of the HI-P PE nanofiber junction as a function of working temperature *T*_1_ and temperature bias Δ*T* with (**a**) the 7.5 K temperature range of phase transition of the pristine segment, and (**b**) the step-function phase transition. The white dashed line marks the local maximum rectification *ε*_*max_local*_ at a certain temperature bias Δ*T*. The length fraction of irradiation is 5%. (**c**) and (**d**) Comparison of heat flow curves with our previous experimental data, for the forward and reverse biases, respectively. The experimental data are measured at working temperature 435 K with a ~ 9 K temperature bias. The phase transition model is modified with phase transition range from 435 to 440 K, based on our experimental data of the pristine PE nanofiber.

In our model, rectification requires that phase transition only occurs in the reverse bias or is less influential in the forward bias as compared to the reverse bias, if phase transition happens in both the biases. Therefore, thermal rectification is always present when the working temperature *T*_1_ is within the range (*T*_1_* − Δ*T*, *T*_2_*). Figure 3a presents the rectification of the HI-P junction as a function of working temperature *T*_1_ and temperature bias Δ*T*. Within 0 < Δ*T* < 10 K, rectification factor increases with increased temperature bias for the whole working temperature range of 430–460 K considered here. Moreover, the results show that the temperature bias is required to be greater than 2 K in order to achieve a rectification factor of > 10%, as shown in Fig. 3a. This is because if a temperature bias is much smaller than the temperature window of phase transition, phase transition will have little influence on rectification. As a comparison, we also investigate the case where the thermal conductance change due to phase transition is simplified by a step function at 447.5 K, as shown in Fig. 3b. In contrast to the results with a finite temperature range (Fig. 3a) for phase transition, Fig. 3b indicates a narrower range of working temperature for effective rectification, but much higher rectification factors. The maximum of rectification factors is no longer achieved at the highest temperature bias but varies with the working temperature *T*_1_. The higher the working temperature is, the lower the optimal temperature bias is. Moreover, the constraint of > 2 K temperature bias for effective rectification is relaxed, and there can always be notable rectification even for an extremely small temperature bias if the working temperature is close to 447.5 K. In Fig. 3c,d, we compare the heat flow curves predicted from the two schemes with our previous experimental results^6^. The temperature bias and heat flow are normalized to their corresponding maximum values. The highest temperature bias is selected to be the same for theoretical and experimental data. Both the step-function and the finite-temperature-range models give almost the same prediction of heat flow for the forward bias, as phase transition does not occur in the forward bias. In contrast, phase transition occurs in the reverse bias. The step-function model depicts the phase transition with a sharp bending of heat flow, while the finite-temperature-range model gives a smooth curve with a better fit of experimental data, as shown in Fig. 3d. Specifically phase transition happens in the range of 435 K to 440 K for the calculation in Fig. 3d, based on our previous experimental study^31^.

**Figure 4:**
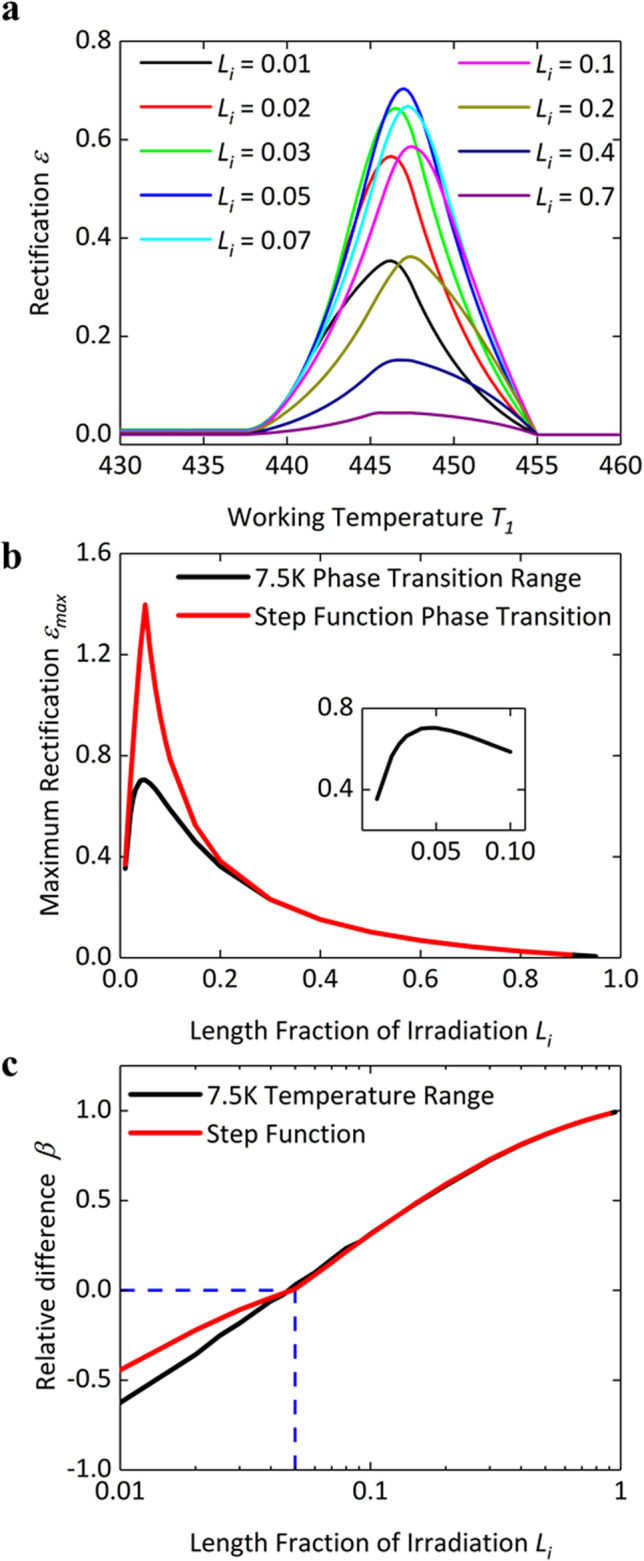
Optimization study of the length fraction of irradiation. (**a**) Rectification as a function of working temperature *T*_1_ with different length fractions of irradiation. (**b**) Maximum rectification *ε*_*max*_ as a function of the length fraction with the 7.5 K temperature range and the step function models for phase transition of the pristine segment. The inset figure shows maximum rectification *ε*_*max*_ as a function of length fraction within the range of 0–0.1 for the finite-temperature-range model. (**c**) Relative temperature difference *β* as a function of the length fraction of irradiation.

To optimize the rectification performance of the HI-P junction, we calculate its rectification factor as a function of the working temperature for different length fractions of e-beam irradiation, as shown in Fig. 4a. No matter how large the length fraction is, there always exists a maximum rectification for the working temperature around the phase transition of the pristine section. In Fig. 4b, we further plot the maxima of rectification factors as a function of the length faction. 5% length fraction of e-beam irradiation leads to the largest rectification based on the thermal conductance data fitted from Fig. 1d. As the thermal conductance of the HI PE nanofiber is extremely low as compared with that of the pristine fiber, it is reasonable that such a short length fraction (5%) gives rise to the largest rectification. Otherwise, the total thermal resistance of the junction is dominated by the HI part and phase transition of the pristine part would not significantly impact the performance of the junction, consequently leading to a smaller rectification value. On the other hand, if the length fraction of e-beam irradiation is too short, the HI-P junction behaves like a pristine fiber and thus has a low rectification factor as well. The rectification performance with the step-function model is also studied for comparison. In Fig. 4b, the step-function model tends to overestimate the rectification performance near the optimal working temperature *T*_1_. However, the two models give almost the same rectification value if the length fraction of irradiation is greater than 30%, as the HI part becomes dominant and the difference of the curve fitting for phase transition becomes minimal. Interestingly, both the two models give almost the same optimal length of fraction. As suggested by the step-function model^15^, rectification is optimized when interface temperatures of the forward and the reverse biases are the same. Here, we calculate the relative temperature difference *β* = (*T*_*i_rev*_ − *T*_*i_fwd*_)*/*Δ*T*, as shown in Fig. 4c. The optimal rectifications predicted by the two models are achieved at the same condition when *β* is equal to zero^15^. Thus, the optimal length fraction of irradiation can still be estimated with formula based on the step-function model^15,30^, even though the rectification factor varies significantly with the value of temperature bias:

5 $$\frac{{L_{A} }}{{L_{B} }} = \frac{{k_{A} }}{{\sqrt {k_{B,1} k_{B,2} } }}$$

where subscripts A and B are the sections without and with phase transition, respectively. B,1 and B,2 represent the phases before and after phase transition, respectively. A and B denote the HI and the pristine sections, respectively, for the HI-P junction, while A or B can be either the LI or the pristine sections, respectively, for the LI-P junction.

**Figure 5:**
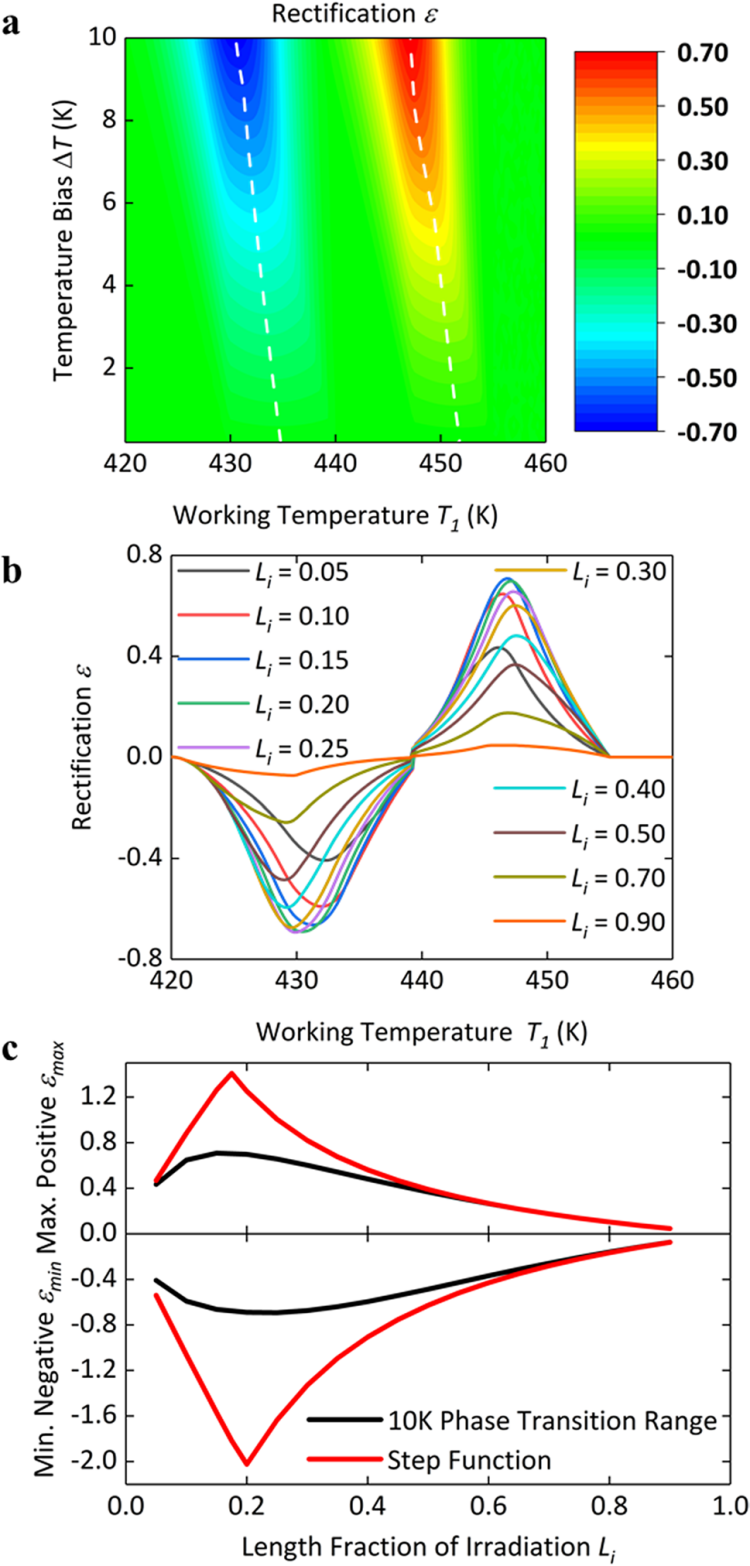
Rectification performance and optimization study of the LI-P junction. (**a**) Rectification performance of the LI-P junction as a function of working temperature *T*_1_ and temperature bias Δ*T.* The length fraction of irradiation is 20%. The white dashed lines mark the local maximum positive rectification *ε*_*max_local*_ and local minimum negative rectification *ε*_*min_local*_ at a certain temperature bias Δ*T*, respectively. (**b**) Rectification as a function of working temperature *T*_1_ with different length fractions of irradiation. (**c**) Maximum positive rectification *ε*_*max*_ and minimum negative rectification *ε*_*min*_ as a function of length fraction with finite temperature ranges of phase transition for both the segments, 10 K for the LI segment and 7.5 K for the pristine segment, and the step function phase transitions for both the segments, respectively.

We also investigate the rectification performance of the LI-P junction based on both the finite-temperature-range and the step-function models. For the LI-P junction, with the definition of rectification factor in Eq. (4), phase transition of the irradiated segment enables negative rectification at a relatively low working temperature while phase transition of the pristine segment induces positive rectification like an HI-P junction at a relatively high working temperature. As shown in Fig. 5a, both the negative rectification at low temperature and the positive rectification at high temperature increase at higher temperature biases and reach the magnitude of 70% at a 10 K temperature bias. Although the thermal switching ratio of the LI segment is higher (~ 9) than that of the pristine segment (~ 5), the minimum temperature bias required for a rectification factor > 10% is still higher than that for the positive rectification, as phase transition of the LI segment occurs within a relatively wider temperature range of ~ 10 K. Similarly, we calculate the rectification ratio with different length fractions of irradiation, as shown in Fig. 5b. Generally, the optimal length fraction of irradiation for negative rectification is not always equal to that for positive rectification. However, it requires these two values to be close to each other for real applications. Here, we use Eq. (5) to estimate the optimal length fractions of irradiation, which are 19.4% and 16.9% for negative and positive rectification, respectively. These two values are close, and consequently the LI-P junction shows negative and positive rectification optimized within the irradiation fraction range from 15 to 25%, as shown in Fig. 5b. We also calculate the optimal length with the step-function model for comparison, as shown in Fig. 5c. Within a 10 K temperature bias, the finite-temperature-range model predicts a flatter rectification curve, that is, the rectification factor is less sensitive to the length fraction of irradiation. In contrast, if the length fraction of irradiation *L*_*i*_ slightly deviates from the optimized value, the rectification factor calculated by the step-function model drops sharply from the maximum value. Moreover, the calculation based on the step-function model shows a larger negative rectification factor than the positive one, as the LI segment has a higher thermal switching ratio than the pristine segment. However, the calculation with the finite-temperature-range model suggests that the larger temperature range of the phase transition of the LI segment compromises the influence of thermal switching ratio. The magnitude of negative rectification is close to that of positive rectification within the whole irradiation length range. Similar with the HI-P junction, as the length fraction of irradiation increases beyond 20%, both positive and negative rectification begin to decrease, because the LI-P junction becomes similar to an LI fiber with no rectification at low working temperatures and the pristine part becomes less effective for positive rectification at high working temperatures. The discrepancy of both positive and negative rectification between the two models also gradually disappears when increasing the length fraction of irradiation.

**Figure 6:**
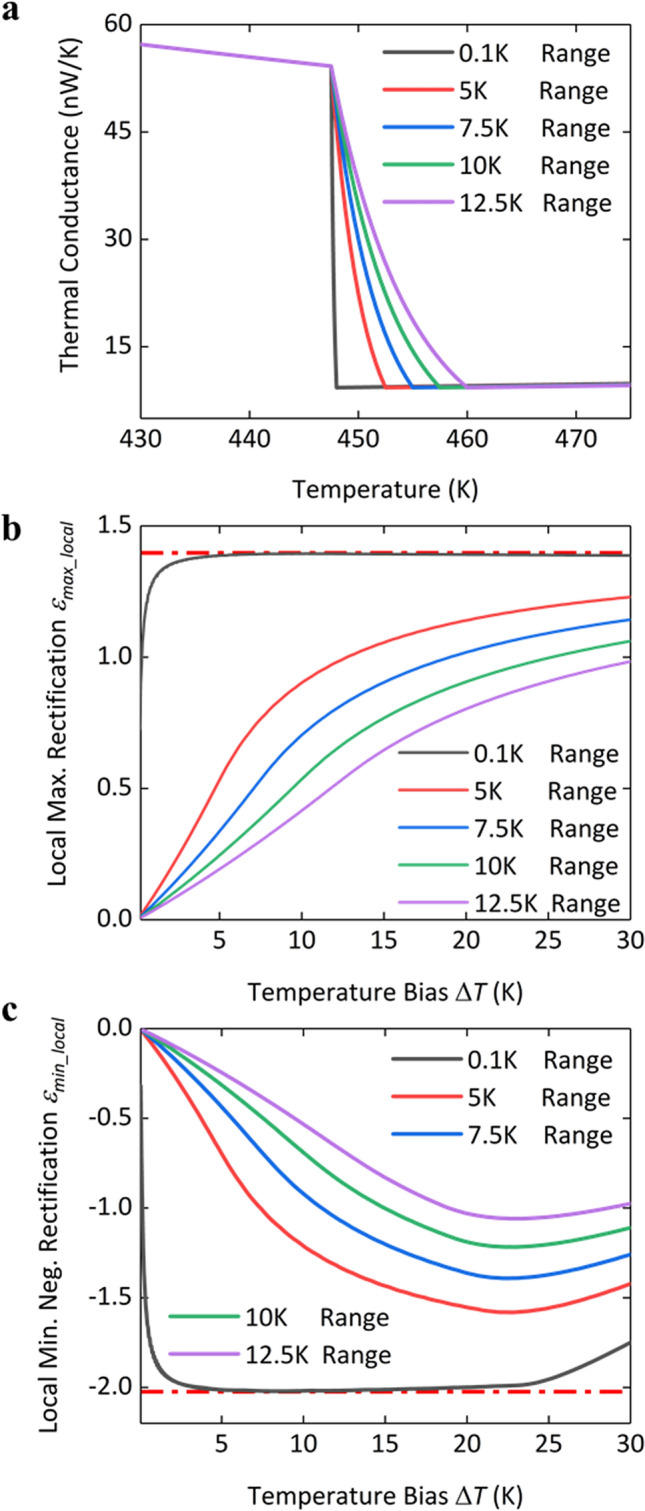
Influence of the temperature range of phase transition on rectification. (**a**) Thermal conductance versus temperature of the pristine segment with a series of temperature ranges of phase transition. (**b**) Local maximum rectification *ε*_*max_local*_ at certain temperature biases within the working temperature *T*_1_ range of 435–455 K of HI-P junctions. The red dash-dotted line shows the maximum rectification indicated by the step-function model. The length fraction of irradiation is 5%. (**c**) The local minimum negative rectification *ε*_*min_local*_ at certain temperature biases within the working temperature *T*_1_ range of 420–455 K of LI-P junctions with the different phase transition temperature ranges of the LI segment. The red dash-dotted line shows the minimum negative rectification indicated by the step-function model. The length fraction of irradiation is 20%.

Lastly, we study the influence of the temperature range of phase transition on the rectification performance. A series of cases are set by varying the temperature range of phase transition of the pristine segment while other parameters including thermal switching ratio and starting temperature *T*_1_^*^ of phase transition are fixed to be the same, as shown in Fig. 6a. We calculate the rectification performances of the corresponding HI-P junctions. The local maximum rectification is plotted in Fig. 6b at certain temperature biases within a range of working temperature *T*_1_. Local maximum rectification factor increases with temperature bias, and asymptotically reaches the upper limit indicated by the step-function model. A larger temperature range of phase transition requires a higher temperature bias for reaching the limit. Similarly, we also vary the temperature range of phase transition of the LI segment in the LI-P junction and calculate the corresponding rectification performances, as shown in Fig. 6c. One unique aspect of LI-P junctions is that phase transition may simultaneously occur in both the LI and the pristine segments, if the temperature bias Δ*T* is so large that *T*_2_ induces the phase transition of the pristine segment. Local minimum negative rectification factor *ε*_*min_local*_ would decrease when increasing temperature bias Δ*T* in such the case. Consequently, rectification factor *ε* may never reach the upper limit under certain working temperature *T*_1_ for LI-P junctions with a large temperature range of phase transition of the LI segment.

## Discussion and conclusion

The thermal rectification of the PE nanofiber junction derives from the asymmetric thermal conductance induced by the phase transition of a pristine or lightly irradiated PE nanofiber. Therefore, the working temperature of the thermal diode is determined by the temperatures of phase transitions, which are greater than 400 K (130 °C) for a PE nanofiber. The thermal diode proposed in this work will enable more applications if the phase transition temperature is close to room temperature. As suggested by the molecular dynamics study^36^, the phase transition temperature of crystalline PE nanofibers can be decreased with softer backbone and weaker interchain interaction. Besides, our study^6^ shows that e-beam irradiation could decrease the phase transition temperature. Therefore, we believe that a lower phase transition temperature can be achieved by chemical modification or precise e-beam irradiation.

A wider temperature range of phase transition can broaden the range of effective working temperature. However, the wider the temperature range of phase transition is, the higher the temperature bias it requires for high rectification ratio. For instance, 50% thermal rectification ratio is realized in the PE nanofiber junction^6^ under a 10 K temperature bias with a 5–10 K temperature range of phase transition, while a 60 K temperature bias is used to realize 43% thermal rectification ratio in the LCO/LSCO junction^16^ with a 60 K temperature range. Generally, the higher the temperature bias is, the more energy is consumed to maintain the temperature bias. Thus, the temperature bias required for notable thermal rectification could be too large and impractical, if the temperature span of phase transition is too wide. In our study, a 10 K temperature bias with a 10 K range of phase transition can effectively balance the requirements of both a wide working temperature range and a small temperature bias.

In summary, we numerically study the junction thermal diodes based on PE nanofibers with phase transition occurring in a finite temperature range. Under different levels of e-beam irradiation, the HI-P junction rectifies heat flow in a single direction, while the LI-P junction shows dual-directional rectification. In contrast with the step-function model, our new model clearly identifies the influence of temperature bias and temperature range of phase transition, that is, rectification generally increases with temperature bias and there is a lower limit for notable rectification. Under a certain temperature bias, a smaller temperature range of phase transition always indicates higher rectification than that of the case with a bigger temperature range. We also optimize the rectification factor by tuning the length fraction of irradiation. Optimized rectification occurs if the interface temperature is the same for both the forward and the reverse biases. The optimal length fraction is about 5% for the HI-P junction. Notable rectification can be realized in both the biases with 15–25% fraction of irradiation for the LI-P junction. Hence, the new model developed in this work can be used to design and predict the rectification performance of phase transition based thermal diodes.

## Data Availability

The data that support the findings of this study are available from the corresponding authors upon request.
